# The role of FGF-2 in smoke-induced emphysema and the therapeutic potential of recombinant FGF-2 in patients with COPD

**DOI:** 10.1038/s12276-018-0178-y

**Published:** 2018-11-14

**Authors:** You-Sun Kim, Goohyeon Hong, Doh Hyung Kim, Young Min Kim, Yoon-Keun Kim, Yeon-Mok Oh, Young-Koo Jee

**Affiliations:** 1 Department of Pulmonary and Critical Care Medicine, Asan Medical Center, University of Ulsan College of Medicine, Seoul, Republic of Korea; 2 Department of Internal Medicine, Dankook University College of Medicine, Cheonan, Republic of Korea; 3 Institute of MD Healthcare, Inc, Seoul, Republic of Korea

## Abstract

Although the positive effects of recombinant fibroblast growth factor-2 (rFGF-2) in chronic obstructive pulmonary disease (COPD) have been implicated in previous studies, knowledge of its role in COPD remains limited. The mechanism of FGF2 in a COPD mouse model and the therapeutic potential of rFGF-2 were investigated in COPD. The mechanism and protective effects of rFGF-2 were evaluated in cigarette smoke-exposed or elastase-induced COPD animal models. Inflammation was assessed in alveolar cells and lung tissues from mice. FGF-2 was decreased in the lungs of cigarette smoke-exposed mice. Intranasal use of rFGF-2 significantly reduced macrophage-dominant inflammation and alveolar destruction in the lungs. In the elastase-induced emphysema model, rFGF-2 improved regeneration of the lungs. In humans, plasma FGF-2 was decreased significantly in COPD compared with normal subjects (10 subjects, *P* *=* 0.037). The safety and efficacy of inhaled rFGF-2 use was examined in COPD patients, along with changes in respiratory symptoms and pulmonary function. A 2-week treatment with inhaled rFGF-2 in COPD (*n* = 6) resulted in significantly improved respiratory symptoms compared with baseline levels (*P* *<* 0.05); however, the results were not significant compared with the placebo. The pulmonary function test results of COPD improved numerically compared with those in the placebo, but the difference was not statistically significant. No serious adverse events occurred during treatment with inhaled rFGF-2. The loss of FGF-2 production is an important mechanism in the development of COPD. Inhaling rFGF-2 may be a new therapeutic option for patients with COPD because rFGF-2 decreases inflammation in lungs exposed to cigarette smoke.

## Lung disease: Inhaling a protein might help

Studies on the role of the protein ‘fibroblast growth factor-2’ (FGF-2) in chronic obstructive pulmonary disease (COPD) suggest that inhaled FGF-2 could help treat the emphysema linked to smoking. Researchers in South Korea led by Young-Koo Jee at Dankook University, Cheonan, and Yeon-Mok Oh at the University of Ulsan, Seoul, studied the role of the reduced FGF-2 levels found in mice with lung inflammation caused by exposure to cigarette smoke. They also uncovered details of a protective effect of inhaled FGF-2, identifying specific cellular and lung structure changes attributed to the administered FGF-2. Reduced FGF-2 levels were also found in patients with COPD. Initial trials revealed some improvement in patients treated with FGF-2, but not at a statistically significant level. Nevertheless, the authors suggest their results justify further investigation of the protein’s therapeutic potential.

## Introduction

Chronic obstructive pulmonary disease (COPD) is a disease entity that is physiologically characterized by chronic irreversible airflow limitation causing progressive decline of lung function and intermittent episodes of increased severity of the disease, known as acute exacerbations. The pathophysiological characteristics of COPD comprise several components, such as mucus hypersecretion, oxidative stress, and chronic inflammation of the airways and lungs^1^. Although bronchodilators, such as long-acting β2-agonists (LABAs) and long-acting muscarinic antagonists (LAMAs), with or without inhaled corticosteroids (ICSs), are recommended as first-line treatments for symptomatic patients, there is no available treatment option to reverse changes in pulmonary structures and declining lung function. In addition, long-term use of corticosteroids can cause complications in patients with advanced stage COPD^2,3^.

The exact mechanism that leads to the presence of pulmonary emphysema, an important part of COPD, is not fully understood. It has been shown that emphysema might be the result of interactions between environmental triggers (such as cigarette smoking) in patients with genetic predisposition. Smoking is known as the main cause of the development of emphysema^4^. Studies have shown that smoking causes an induction of several epigenetic changes in the methylation of DNA, which has been found to occur in emphysema^5^. In the case of emphysema, oxidative stress mainly caused by cigarette smoking results in the development of cellular injury and apoptosis^6^. A common mediator in emphysema is transforming growth factor-β (TGF-β). Although TGF is known as a major regulator of fibrosis, it has recently been found to play a significant role in the development of emphysema^7^. FGF signaling regulates TGF-β-dependent smooth muscle cell phenotypes^8^. In other words, FGF-2 is a downstream mediator of TGF-β and is important in wound healing^9^.

Fibroblast growth factors (FGFs) are members of the heparin-binding growth factor family that affect the growth and differentiation of various cell types^10^. They are often involved in morphogenesis, wound repair, inflammation, and angiogenesis and function as potent chemotactic and mitogenic factors for cells originating from the mesoderm, ectoderm and endoderm^11–14^. In addition, FGF-2 has attracted increasing attention as an important factor involved in airway remodeling by increasing the deposition of proteoglycans and its eventual association with bronchial hyperresponsiveness in asthmatic airways^12–15^.

Previous studies have suggested that there are beneficial effects of recombinant FGF-2 (rFGF-2) in treating COPD in animal emphysema models^16–19^. In addition, rFGF-2 has been reported to be an inhibitor of inflammatory cells and cytokines related to asthma and has been shown to reduce airway remodeling in an asthma mouse model^20^. In a clinical trial, inhaled rFGF-2 was safely used in patients with asthma, whose daytime and nocturnal symptoms as well as quality of life were improved without any serious adverse events (AEs)^21^. Regardless of these positive results of rFGF-2 for treating animal emphysema models, it is reasonable to assume that rFGF-2 would be effective in patients with COPD based on the similarity between the pathogeneses of COPD and asthma. Furthermore, the development of fundamental medicine to prevent airway hyperresponsiveness and airway remodeling in patients with COPD has been considered a priority.

In this study, the mechanisms of FGF-2 in the development of COPD (especially emphysema) and the therapeutic potential of FGF2 were explored in a cigarette smoke-exposed C57BL/6 J emphysema mouse model. Additionally, a prospective placebo-controlled pilot study was conducted separately to define the potential role of rFGF-2 in improving or stabilizing symptoms and lung function in patients with COPD.

## Materials and Methods

### Animal experiments

#### Animal model

C57BL/6 J mice were purchased from Orientbio (Seongnam, Korea). The mice were bred in specific pathogen-free facilities at Asan Medical Center. Female C57BL/6 J mice (7 weeks of age) were exposed to commercial cigarette smoke based on the protocol used in our previous report^22^.

Briefly, mice were exposed to smoke for 1 day, 4 days, and 6 months (5 days/week) in an inhalation box (50 × 40 × 30 cm) connected to a pump to assess changes in FGF-2 levels. To evaluate the effect of short-term smoke exposure on the mice, mice were exposed to smoke for 4 days, and 0.1, 10, or 1,000 pg of rFGF-2 was intranasally administered to each mouse immediately after smoke exposure on each day to define the effects of rFGF-2 on smoke exposure. After completing the 4-day smoke exposure with or without rFGF-2, the mice were killed to collect their lung tissues on day 5.

For the elastase-induced mouse model, 0.4 U of porcine elastase (Sigma-Aldrich, St. Louis, MO, USA) was intratracheally injected into C57BL/6 mice on day 0. Then, rFGF-2 (10 pg) was administered intranasally once daily from days 7–13, and PD173074 was intraperitoneally injected once per day during the same period. The mice were killed to collect their lung tissues on day 14.

All mouse experiments were approved by the Institutional Animal Care and Use Committee of Asan Medical Center (South Korea).

#### Bronchoalveolar lavage cellularity

Cellularity in Bronchoalveolar lavage (BAL) fluid was analyzed as previously described^23^. In brief, BAL fluid was obtained from both lungs using PBS, and the cells were collected after centrifugation. The total number of cells in the BAL fluid was counted after trypan-blue staining, and the cell pellet was diluted with 500 µL of PBS for counting. The BAL differential cell count was determined by Diff-Quick staining in 300 inflammatory cells counted. Inflammatory cells were classified as macrophages, lymphocytes, neutrophils or eosinophils.

#### Histology and quantification of emphysema

The lungs were perfused with PBS and inflated by intratracheal infusion of 0.5% low-melting agar in 25 cm H_2_O, fixed in 4% paraformaldehyde and embedded in paraffin. Lung sections of 4-μm thickness were stained with hematoxylin and eosin (H&E). The mean linear intercept (MLI) was determined separately by 2 investigators in a blinded fashion^22^.

#### Caspase-3/7 activity and western blotting

A 10-μg portion of lung protein homogenized in RIPA buffer (Cell Signaling Technology, Danvers, MA, USA) was measured with the Apo-one homogeneous caspase-3/7 assay kit (Promega, Madison, WI, USA) according to the manufacturer’s protocol. After 5 h, sample fluorescence was measured using a fluorometer. Western blotting using anti-caspase-3 polyclonal antibody (Enzo Life Science, New York, NY, USA) was performed to quantify pro- and active caspase-3 in lung tissues.

#### Elastase activity

Lung tissues were homogenized with protein lysis buffer (Cell Signaling Technology) without protease inhibitors and subjected to the EnzCheck Elastase Assay Kit (Molecular Probes, Sunnyvale, CA, USA) according to the manufacturer’s protocol.

#### FGF-2

FGF-2 was measured in the plasma of normal controls and patients with COPD (10 subjects/group). The lungs of mice were homogenized in RIPA buffer, and the quantity of protein was assessed with the Bradford assay. FGF-2 levels were measured using an enzyme-linked immunosorbent assay (ELISA) (R&D Systems, Minneapolis, MN, USA) according to the manufacturer’s protocol.

#### Clinical trial: a pilot study

This single-center, double-blind, randomized, placebo-controlled pilot trial was conducted at Dankook University Hospital between October 2009 and September 2010. The study protocol was approved by the Korean Food and Drug Administration and the Institutional Review Board at Dankook University Hospital (DKUH 0909-066). Written informed consent was obtained from all patients before they participated in the study. Dong-A Pharm. Co. Ltd. (Seoul, South Korea) provided the rFGF-2. The dosage used in this study was determined based on a previous clinical trial performed in patients with asthma^21^.

Randomization was performed using a random number table on day 1 (screening visit). Eligible patients with COPD were randomly allocated to 4.5 ng/day rFGF-2 or a placebo. In the treatment group, 1.5 ng of rFGF-2 was administered over 10 min, 3 times a day for 2 weeks using a nebulizer kit (PARI BOY N® type 085; PARI GmbH, Starnberg, Germany), and normal saline was administered to the placebo group using the same method for the same duration. The biological half-life of FGF is <50 min^24^, which is too short for a single administration by inhalation to be sufficient because wound healing takes longer than 1 day^25^. Therefore, administration 3 times a day was recommended for this clinical pilot trial. Concomitant use of ICSs, LABAs/LAMAs, mucus-active agents, and antitussives was permitted during the study, but use of systemic corticosteroids was prohibited to avoid any potential confounding effects on the clinical endpoints.

Twelve patients with COPD were randomly assigned to a 2-week treatment period (*n* = 6) or placebo (*n* = 6), followed by a 4-week nontreatment follow-up period. Patients were first seen at a screening visit and afterwards at 7, 15, and 28 days after completion of the trial. At screening and at each visit, the following results were recorded: physical examination including vital signs, pulmonary function tests (PFTs), chest radiography, and electrocardiogram (ECG); and blood chemistry, urinalysis, and dyspnea scale including the American Thoracic Society (ATS) dyspnea scale and the modified Borg scale. Patients had to meet all the criteria included in Table S2 in the Supplementary Appendix.

Patients were not eligible for the trial if they met 1 or more of the exclusion criteria listed in Table S3. The clinical characteristics are displayed in Table S3. The primary endpoint was confirmation of the safety of inhaled rFGF-2. Safety was assessed by reporting AEs, vital signs, chest radiography, ECG, and laboratory test results, including complete blood count, blood chemistry, and urinalysis. The secondary endpoints were the clinical effects of rFGF-2 from baseline to 4 weeks of treatment. The clinical effects were assessed by analyzing PFT results, such as changes in forced expiratory volume in 1 s (FEV1), forced vital capacity (FVC), the Global Chest Symptoms Questionnaire (GCSQ)^26^, as well as dyspnea scales including the ATS dyspnea scale and the modified Borg scale.

Data are presented as the mean and standard deviation for continuous variables and as a number (percentage) for categorical variables. The Mann–Whitney *U* test was used to compare plasma FGF-2 levels between the COPD and control groups. Two-way analysis of variance (ANOVA) was performed for BAL cellularity. In the human clinical study, differences between the study and control groups were tested using the Wilcoxon matched pair signed-rank test. A *P* value of <0.05 was considered statistically significant. The data were analyzed using PASW statistics ver. 18 (SPSS Inc., Chicago, IL, USA).

## Results

### FGF-2 production in the lungs is dependent on exposure time to cigarette smoke

**Fig. 1:**
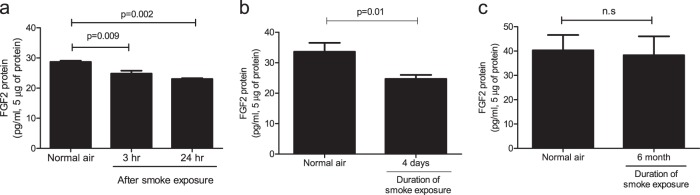
Fibroblast growth factor-2 (FGF-2) levels in lung lysates decreased in response to short-term smoke exposure. **a**–**c** FGF-2 levels were measured in 5 μg of lung lysate using an enzyme-linked immunosorbent assay. **a** Mice were exposed to cigarette smoke once, and lung tissues were obtained 3 and 24 h after exposure (*n* = 6 per group). **b**, **c** Mice were exposed to cigarette smoke for 4 days or 6 months, and lung tissues were collected 18 h after the last exposure (**b** *n* = 12 per group, **c** *n* = 5 per group). *P-*values were determined by the Mann–Whitney test

To evaluate the effects of exposure to cigarette smoke on FGF-2 production in the lungs, the amounts of FGF-2 in lung lysates were compared using ELISA in C57BL/6 mice. Compared with mice exposed to normal air, FGF-2 production in lung tissues was decreased 3 and 24 h after smoke exposure in mice exposed to smoke once (Fig. 1a). A decrease in FGF-2 levels was also observed in mice exposed to smoke for 4 days (Fig. 1b). However, the level of FGF-2 produced in mice exposed to smoke for 6 months was not significantly different from that in mice exposed to normal air (Fig. 1c). Therefore, FGF-2 production was decreased in mouse lungs only after short-term exposure to cigarette smoke.

### Emphysema induced by short-term exposure to cigarette smoke in mice

**Fig. 2:**
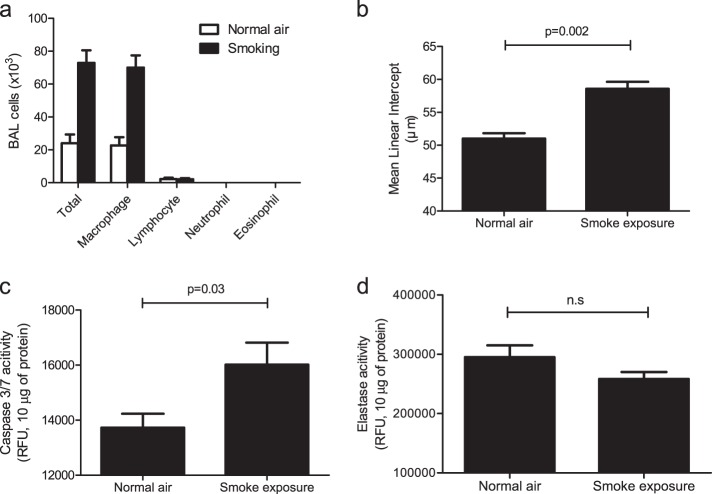
Characterization of short-term exposure to cigarette smoke. **a**–**d** The mice were exposed to cigarette smoke for 4 days using an inhalation box. **a** Cellularity in bronchoalveolar lavage (BAL) fluid. *P* < 0.0001 using two-way analysis of variance. **b** Mean linear intercept (MLI) (*n* = 6 per group). **c** Caspase-3/7 and elastase activities were measured in 10 μg of lung lysate (*n* = 11 per group). *P*-values were determined by the Mann–Whitney test (**b**–**d**)

To determine the effects of a decreased FGF-2 level on the lungs after short-term exposure to cigarette smoke (presented in Fig. 1), BAL fluid and lung tissue were collected on day 5 from C57BL/6 mice exposed to cigarette smoke for 4 days, and inflammation and the emphysema index representing the COPD patient’s phenotype were determined. Recruitment of inflammatory cells was increased in smoke-exposed mice; macrophages were the main inflammatory cell type that was increased in the BAL fluid of the smoke-exposed mice (Fig. 2a). The MLI was measured using H&E-stained lung tissue to determine the index of alveolar destruction, which was significantly increased in smoke-exposed mice compared with that in normal air-exposed mice (Fig. 2b). Lung resident cell apoptosis and protease activity developed in the destroyed alveoli. Apoptosis based on caspase-3/7 signaling in lung tissue was increased more in mice exposed to cigarette smoke than in those exposed to normal air (Fig. 2c), but alveolar destruction observed in smoke-exposed mice was not associated with an induction of elastase activity (Fig. 2d). Overall, mice that were exposed to short-term smoke showed phenotypes similar to those of patients with COPD, such as macrophage-dominant inflammation and alveolar destruction.

### Protective effect of rFGF-2 on the development of emphysema induced by exposure to short-term cigarette smoke in mice

**Fig. 3:**
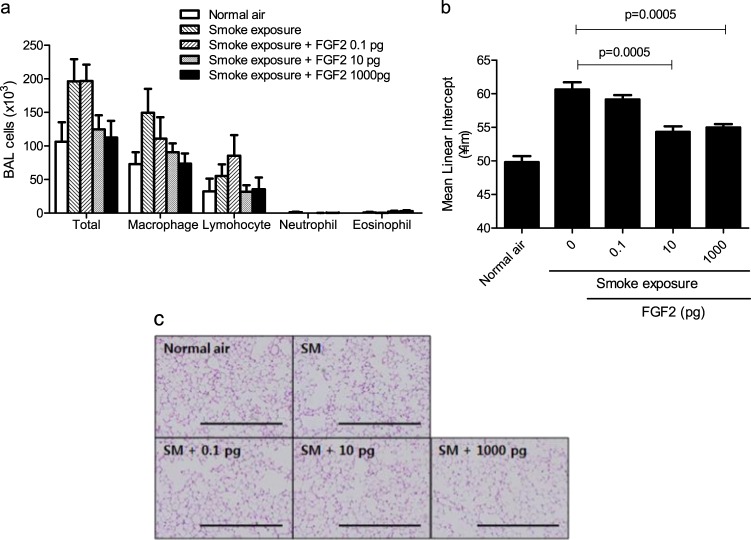
Protective effects of FGF-2 on early emphysema in response to short-term exposure to cigarette smoke. **a**–**c** The mice were exposed to cigarette smoke for 4 days, and 0.1, 10, and 1000 pg of FGF-2 was administered intranasally within 1 h after smoke exposure. Mice were killed 18 h after the final smoke exposure. **a** Cellularity in BAL fluid. *P*-values were determined by two-way analysis of variance. **b** MLI (*n* = 6–12 per group). *P*-values were determined by the Mann–Whitney test. **c** Lung histology with hematoxylin and eosin (H&E) staining (scale bar = 1 mm)

To confirm the protective effect of rFGF-2, 3 doses (0.1, 10, and 1000 pg) of the rFGF-2 protein were intranasally administered immediately after the mice were exposed to smoke for 4 days. The number of inflammatory cells recruited was decreased more in mice administered 10 or 1000 pg of rFGF-2 than in those administered 0 or 0.1 pg of rFGF-2 after smoke exposure (Fig. 3a). In particular, the numbers of macrophages and lymphocytes were decreased in BAL fluid obtained from the 10 and 1000 pg rFGF-2-administered mice. Alveolar destruction was also decreased in mice administered 10 and 1000 pg of rFGF-2 (Fig. 3b, c). These results suggest that the loss of FGF-2 production is an important initial mechanism of developing COPD in response to cigarette smoke, and this phenomenon was protected by and compensated for using rFGF-2.

### Effect of rFGF-2 on apoptosis signaling in cigarette smoke-exposed mice

**Fig. 4:**
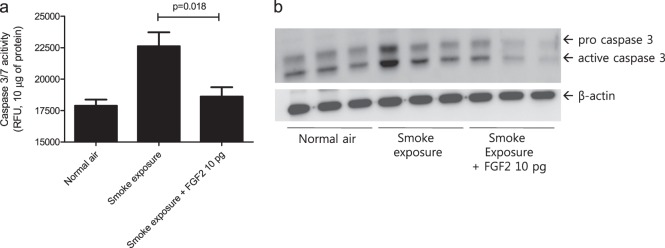
Lung resident cell apoptosis in response to smoke was protected against by FGF-2 and dependent on caspase-3/7 signaling. **a**, **b** The mice were exposed to cigarette smoke for 4 days, and 10 pg of FGF-2 was intranasally administered within 1 h after smoke exposure. Mice were killed 18 h after the final smoke exposure. **a** Caspase-3/7 activity using 10 μg of lung lysate (*n* = 9 per group). *P*-values were determined by the Mann–Whitney test. **b** Western blotting using antibodies against caspase-3 and β-actin

Resident lung cell apoptosis developed in the destroyed alveoli of mice exposed to smoke over the short term (Fig. 2). Resident lung cell apoptosis was dependent on caspase-3 signaling in mice exposed to smoke for 4 days. Caspase-3/7 activity was significantly decreased immediately after smoke exposure in mice intranasally administered 10 pg of rFGF-2 each day than in those exposed to smoke alone (Fig. 4a). Pro- and active caspase-3 protein expression was also decreased in the group receiving rFGF-2 (Fig. 4b).

### Regenerative effect of rFGF-2 on the development of emphysema in the elastase-induced mouse model

**Fig. 5:**
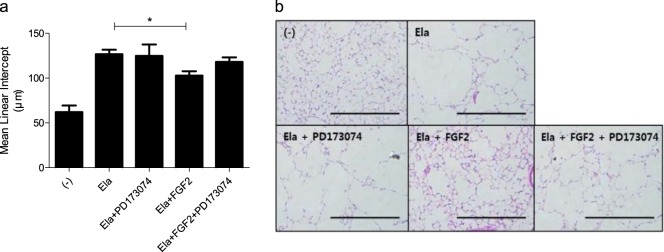
Regenerative effects of FGF-2 on the elastase-induced emphysema model. The mice were intratracheally injected with 0.4 U of porcine elastase on day 0. Aliquots (10 pg) of FGF-2 and PD173074 were intranasally and intraperitoneally administered on days 7–13. The mice were killed on day 14. **a** MLI (*n* = 4–6 per group). *P*-values were determined by the Mann–Whitney test; *p < 0.05. **b** Lung histology with H&E staining (scale bar = 1 mm)

To evaluate the regenerative effects of FGF-2, rFGF-2 was intranasally administered to mice with emphysema, which was induced by injecting porcine elastase for 7 days. The rFGF-2 protein (10 pg) was administered once daily for 4 days after completing the elastase injection schedule. The lungs were effectively regenerated in rFGF-2-treated mice compared with nontreated mice (Fig. 5a, b). To confirm the direct association between emphysematous lung regeneration and FGF-2, we used PD173074 as an FGF receptor 1/3 inhibitor. The regenerative effect shown in elastase-induced emphysematous mice receiving rFGF-2 was not observed when PD173074 was given intraperitoneally (Fig. 5a, b), suggesting a direct association between FGF-2-mediated signaling and the regeneration of emphysematous lungs.

### Blood FGF-2 levels in patients with COPD

**Fig. 6:**
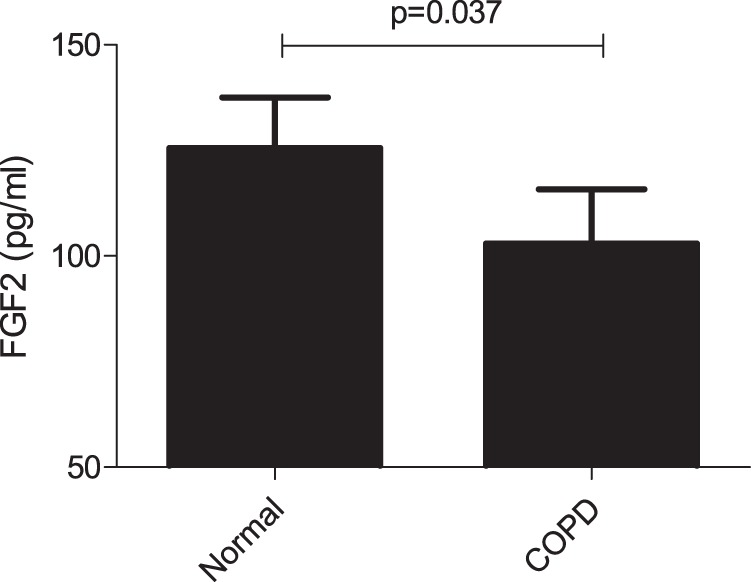
FGF-2 levels in plasma from patients with chronic obstructive pulmonary disease (COPD) and normal controls. A 100-µL aliquot of plasma was used to measure FGF-2 (*n* = 10/group). *P* = 0.037 according to the Mann–Whitney test

To determine whether the blood FGF-2 level is correlated with COPD, FGF-2 levels were measured in plasma from patients with COPD and normal controls using ELISA in 10 subjects each from the study groups. Plasma was obtained from individuals with similar ages and histories (Table S1). The mean plasma FGF-2 level was 102.9 pg/mL in patients with COPD and 125.5 pg/mL in the normal controls (*P* = 0.037) (Fig. 6).

### Safety of inhaling FGF-2 in patients with COPD

**Fig. 7:**
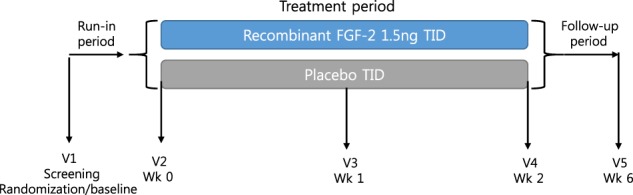
Flowchart of the clinical pilot trial. V visit; Wk week

The flowchart for the clinical pilot trial is shown in Fig. 7. No treatment-associated severe AEs requiring treatment discontinuation were observed in the rFGF-2 group. No abnormal findings were detected on physical examination in the treatment group during the study period. Petechiae were found in 1 patient in the control group on day 2 of rFGF-2 treatment, but the lesions disappeared without any medication. No abnormal findings were observed on chest radiographs, ECGs or laboratory tests, including a complete blood count, blood chemistry, and urinalysis, during the study period.

The most commonly reported AEs were cough and sore throat. The incidences of AEs are summarized in Table S4. The AEs improved spontaneously during treatment with inhaled rFGF-2. Therefore, AEs were not causally related to inhaled rFGF-2 treatment.

### Clinical effects of inhaling FGF-2 in patients with COPD

**Fig. 8:**
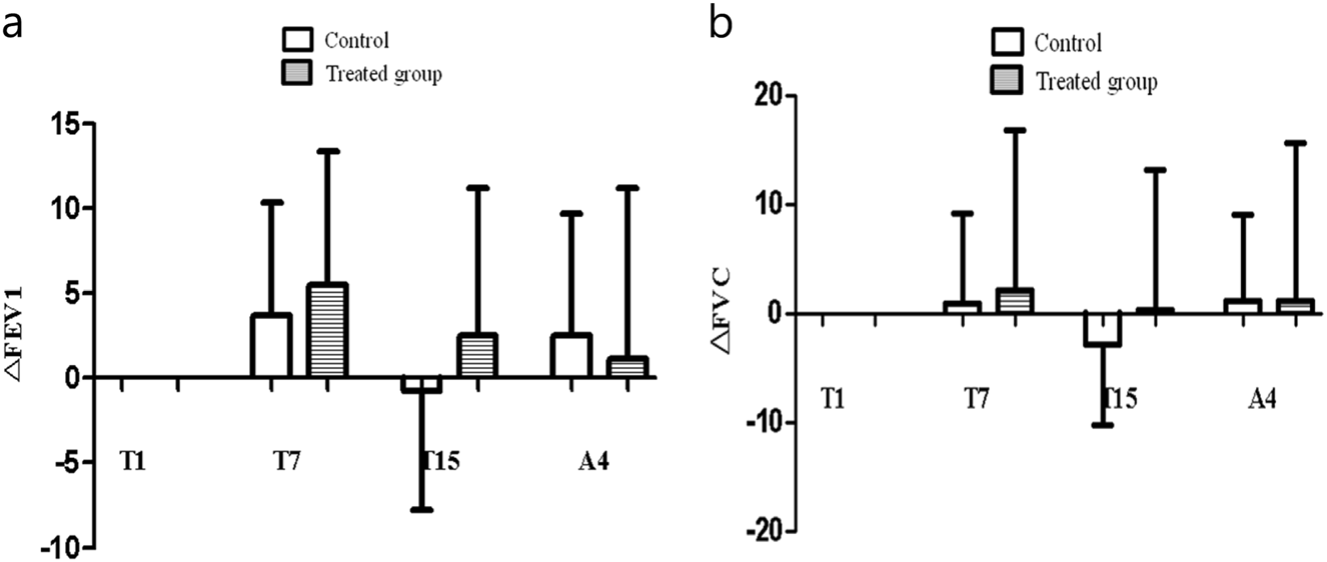
Change in pulmonary function tests (PFTs) [**a** change in forced expiratory flow in 1 s (Δ FEV_1_) and **b** change in forced vital capacity (Δ FVC)] during and after fibroblast growth factor (FGF)-2 treatment. PFTs, including FEV1 and FVC, tended to improve during the recombinant (r) FGF-2 treatment period but not significantly between the rFGF-2 group and the control group (*p* > 0.05)

All patients enrolled in the clinical trial were current smokers. The demographic characteristics of patients are summarized in Table 1. PFTs including FEV1 and FVC tended to improve during the rFGF-2 treatment period, but they were not significantly different between the rFGF-2 and control groups *P* *>* 0.05. Changes in the PFTs are summarized in Table 2 and Fig. 8.

**Table 1:** Demographic characteristics of the subjects in the clinical trial

	Control group (*n* = 6)	FGF-2 group (*n* = 6)	*p*-value^a^
Age (year)	66.7 ± 11.7	63.0 ± 10.5	0.39
Male (%)	6 (100)	5 (83)	0.30
BMI (kg/m^2^)	21.8 ± 3.3	23.3 ± 3.6	0.39
Smoking pack-years	45.0 ± 10.9	32.0 ± 17.3	0.24
*Pulmonary function tests*			
FEV_1_ (%)	41.6 ± 9.19	49.7 ± 7.4	0.24
FVC (%)	63.6 ± 10.5	74.2 ± 15.2	0.13

*FGF-2* fibroblast growth factor-2, *BMI* body mass index, *FEV*_*1*_ forced expiratory volume in 1 s, *FVC* forced vital capacity

^a^Statistical differences were tested by Pearson’s chi-square test

**Table 2:** Pulmonary function test (FEV_1_/ΔFEV_1_ and FVC/ΔFVC) of each group during and after FGF-2 treatment in the clinical trial

	FEV_1_ (%)		ΔFEV_1_ (%)		FVC (%)		ΔFVC (%)	
	Control	FGF-2	Control	FGF-2	Control	FGF-2	Control	FGF-2
T1	42.8 ± 9.7	49.7 ± 7.4	0.0 ± 0.0	0.0 ± 0.0	65.2 ± 10.9	74.2 ± 15.2	0.0 ± 0.0	0.0 ± 0.0
T7	46.6 ± 13.6	55.2 ± 5.3	3.7 ± 6.6	5.5 ± 7.8	66.0 ± 17.7	76.3 ± 9.9	0.9 ± 8.3	2.1 ± 14.7
T15	42.1 ± 12.5	52.3 ± 5.9	−0.7 ± 7.1	2.5 ± 8.7	62.4 ± 15.1	74.5 ± 11.3	−2.8 ± 7.5	0.3 ± 12.9
A4	45.4 ± 8.3	50.9 ± 6.8	2.5 ± 7.2	1.1 ± 10.1	66.3 ± 10.3	75.3 ± 8.5	1.1 ± 7.9	1.1 ± 14.6

*FEV*_*1*_ forced expiratory volume in one second, *FVC* forced vital capacity, *FGF-2* fibroblast growth factor-2, *T1* 1 day of treatment, *T7* 7 days of treatment, *T15* 15 days of treatment, *A4* after 4 weeks of treatment

According to the GCSQ results, the grade of dyspnea, chest discomfort, and daytime activities improved more after treatment than before treatment in the rFGF-2 group (*P* *<* 0.05). The ATS dyspnea scale score improved significantly 4 weeks after treatment compared with the first day of treatment (*P* *=* 0.038). The modified Borg scale score improved significantly 4 weeks after treatment (2.5 ± 1.4) compared with the first day of treatment (4.8 ± 2.0) and day 7 of treatment (4.0 ± 1.1) (*P* *=* 0.042 and *P* *=* 0.041, respectively). Chest discomfort improved significantly on day 15 of treatment (0.3 ± 0.5) compared with the first day of treatment (1.7 ± 1.0) (*P* *=* 0.038). Daytime activities significantly improved more during the 4 weeks after treatment (12.8 ± 8.6) than during the first day of treatment (21.8 ± 9.7) and day 15 of treatment (28.3 ± 14.8) (*P* *=* 0.042 and *P* *=* 0.043, respectively). In particular, the effects of rFGF-2 were maintained for up to 4 weeks after treatment. However, no significant differences were observed between the rFGF-2 and control groups for GCSQ, grade of dyspnea, chest discomfort, or daytime activities (*P* *>* 0.05). The dyspnea scales, including the ATS dyspnea scale and the modified Borg scale, were not significantly different between the rFGF-2 and control groups. These results are summarized in Tables 3 and 4.

**Table 3:** ATS scale and ΔATS scale of each group during and after FGF-2 treatment in a clinical trial

	ATS scale		ΔATS scale	
	Control	FGF-2	Control	FGF-2
T1	2.2 ± 0.4	2.2 ± 0.8	0.0 ± 0.0	0.0 ± 0.0
T7	1.6 ± 1.1	2.2 ± 0.8	−0.6 ± 0.9	0.0 ± 0.0
T15	1.8 ± 0.8	2.0 ± 0.0	−0.4 ± 0.5	−0.2 ± 0.8
A4	1.4 ± 0.9	1.5 ± 0.8	−0.8 ± 0.4	−0.7 ± 0.8

*ATS* American Thoracic Society, *FGF-2* fibroblast growth factor-2, *T1* 1 day of treatment, *T7* 7 days of treatment, *T15* 15 days of treatment, *A4* after 4 weeks of treatment.

**Table 4:** Modified Borg scale and ΔModified Borg scale of each group during and after FGF-2 treatment in a clinical trial

	Modified Borg scale		ΔModified Borg scale	
	Control	FGF-2	Control	FGF-2
T1	4.0 ± 1.2	4.8 ± 2.0	0.0 ± 0.0	0.0 ± 0.0
T7	3.2 ± 1.9	4.0 ± 1.1	−0.8 ± 1.3	−0.8 ± 1.2
T15	2.6 ± 1.8	3.3 ± 0.5	−1.4 ± 1.1	−1.5 ± 1.9
A4	2.6 ± 2.1	2.5 ± 1.4	−1.4 ± 1.1	−2.3 ± 1.9

*FGF-2* fibroblast growth factor-2, *T1* 1 day of treatment, *T7* 7 days of treatment, *T15* 15 days of treatment, *A4* after 4 weeks of treatment.

## Discussion

This study revealed several important findings. First, plasma FGF-2 levels were decreased more in patients with COPD than in normal subjects. Second, FGF-2 levels were decreased in short-term smoke-exposed mice. Finally, FGF-2 significantly protected against the development of emphysema induced by short-term exposure to cigarette smoke in mice.

A significant inverse correlation was observed between short-term exposure to cigarette smoke and FGF-2 production, indicating that the degree of pulmonary FGF-2 expression is related to the magnitude of lung exposure to smoke during the early phase. In this study, we tested very short-term exposure to smoke in animal experiments. Biological changes induced by smoke mainly develop during the early phase; therefore, biological changes are not clearly observed in the late phase. The mechanisms involved in this phenomenon are poorly understood. In fact, FGF-2 levels changed upon short-term exposure to smoke. Therefore, we believe that a short-term exposure animal model is effective for examining the effects of FGF-2 inhalation.

In this study, BAL fluid demonstrated macrophage-dominant inflammation and alveolar destruction of lung tissues according to the MLI. An increase in the number of inflammatory cells, mainly macrophages, was seen in the BAL fluid of smoke-exposed mice. Cigarette smoke is a well-defined common pollutant that influences cellular components in BAL fluid^27,28^.

Smokers have a significantly elevated number of cells in the lower respiratory tract, mainly due to the increased number of alveolar macrophages^29,30^. Macrophages are the first line of defense against inhaled pollutants, including tobacco smoke. The MLI, which is the standard technique for measuring alveolar destruction, was significantly increased in smoke-exposed mice compared with normal air-exposed mice.

Intranasal FGF-2 administration had a protective effect on emphysema: (1) a decline in the number of inflammatory cells, especially macrophages and lymphocytes, in BAL fluid; and (2) a decline in alveolar destruction and a regenerative effect in emphysematous mice. These results indicate that loss of FGF-2 production is an important mechanism for developing COPD in response to smoke. Caspase-3/7 activity, which is related to lung cell apoptosis, was significantly decreased in the mice after FGF-2 treatment. Previous studies have shown that FGF-2 prevents apoptosis induced by chemotherapy in patients with lung cancer^31,32^. Intravascular FGF-GMS administration reduces dilatation of the alveolar space and microvascular proliferation in the emphysematous canine lung^16^. Intratracheal administration of FGF-2 increases blood flow in damaged lungs and improves blood gas values, thus allowing recovery of pulmonary function in a canine emphysema model^17,18^. Intrapleural administration of FGF-2 in rats with emphysematous lungs induces lung regeneration^19^. These studies suggest a new potential emphysema treatment using FGF-2, and the present study confirmed these findings. However, the positive effects of FGF-2 in animal studies may not be equally applicable to human COPD. Therefore, we further studied human patients with COPD to determine whether FGF-2 is involved in the pathogenesis of COPD.

In our study, the analysis of FGF-2 in human COPD showed decreased plasma levels of FGF-2 in patients with COPD compared with normal subjects. The mean plasma FGF-2 level was 102.9 pg/mL in patients with COPD and 125.5 pg/mL in the healthy controls (*P* = 0.037). When we consider these marginal differences in FGF-2 levels, other factors involved in the development of COPD should be considered. However, when considering the results of animal experiments together, there might be a protective effect of FGF-2, although FGF-2 levels between COPD patients and healthy controls were marginal. Our findings of downregulated FGF-2 indicate that the compensatory mechanisms observed in mice are also active in patients with COPD, as smoking has been suggested to have a strong effect on the imbalance of proteases/antiproteases, including elastases and collagenases, and extracellular matrix deposition in the lungs. FGF-2 in the bronchial epithelium could be involved in the proliferation and repair of epithelial cells after injury in patients with COPD. However, FGF-2 could also be involved in fibrosis and tissue remodeling in patients with asthma and COPD^33^.

Airway remodeling is an important pathophysiological feature of COPD as well as asthma and is characterized by thickening of the airway wall with increased collagen deposition and airway smooth muscle hypertrophy and hyperplasia^34,35^. The mechanisms underlying airway remodeling are not well understood. One molecule with possible relevance is FGF-2, which is a potent mitogen of fibroblasts, airway smooth muscle cells, and endothelial cells. The degree of airway wall thickening is associated with disease progression, which is the main cause of decreased lung function in patients with COPD because remodeling reduces airflow and distensibility^36–38^. FGF-2 plays an important role in wound healing by promoting the migration and proliferation of fibroblasts, reversing myofibroblast phenotypes, and regenerating airway epithelial cells^11,39^. In particular, FGF-2 has an inhibitory effect on the differentiation of fibroblasts into myofibroblasts, suggesting a possible anti-scarring effect of FGF-2 during wound healing.

Furthermore, we conducted a clinical trial in patients with COPD to determine the effect and safety of inhaled rFGF-2. The results showed that inhaled rFGF-2 was safe and well tolerated in patients with COPD. No significant differences were observed in terms of AEs associated with the inhaled rFGF-2 treatment compared with the placebo during the study period. Only 2 patients developed minimal side effects of cough and sore throat, which were self-limiting.

The present clinical study had several limitations. First, the inclusion of a small number of patients from a single center showed the potential for selection bias. Despite this limitation, this pilot clinical trial is the first one done in humans as a preclinical study. Further large-scale clinical studies are warranted. Second, objective airway inflammatory markers, including inflammatory cells in the sputum, cytokines, and chemokines, were not measured in the clinical trial. However, when the animal experimental results are considered together, rFGF-2 may be effective at suppressing inflammatory changes. Third, there is a paucity of data on the safety, efficacy, and pulmonary pharmacokinetics of rFGF-2 administered via the inhalation route. We were unaware of the concentrations or distribution of rFGF-2 in the pulmonary system. Fourth, our study did not assess long-term safety or patient outcomes, such as the exacerbation rate after completion of treatment. However, clinical improvements and no serious AEs 4 weeks after treatment could be considered reasonable treatment goals for COPD. Finally, we did not evaluate correlations between FGF-2 and lung function/clinical parameters. Large-scale clinical trials should be done in the future.

COPD remains an inextirpable disease with significant morbidity and mortality rates when accompanied by acute exacerbations. Unfortunately, no fundamental treatment agent has been identified. Currently, several drugs targeting the small airways of patients with COPD have been developed and successfully used, but there are no effective drugs for emphysema. The results of this study demonstrate that FGF-2 is involved in the pathogenesis of emphysema; therefore, rFGF-2 is a therapeutic option for Korean patients with emphysema-type COPD. The potential attenuation of symptom progression associated with COPD reported in this study makes rFGF-2 a reasonable agent for further prospective trials.

## Electronic supplementary material

Table S1, Table S2, Table S3, Table S4

## References

[1] Global Strategy for the Diagnosis, Management and Prevention of COPD, Global Initiative for Chronic Obstructive Lung Disease (GOLD) 2018. Available at: http://www.goldcopd.org/.

[2] Wedzicha JA (2016). Indacaterol-glycopyrronium versus salmeterol-fluticasone for COPD. N. Engl. J. Med.

[3] Janson C (2013). Pneumonia and pneumonia related mortality in patients with COPD treated with fixed combinations of inhaled corticosteroid and long acting β2 agonist: observational matched cohort study (PATHOS). BMJ.

[4] Morse D, Rosas IO (2014). Tobacco smoke-induced lung fibrosis and emphysema. Annu Rev. Physiol..

[5] Talikka M (2012). Genomic impact of cigarette smoke, with application to three smoking-related diseases. Crit. Rev. Toxicol..

[6] MacNee W (2000). Oxidants/antioxidants and COPD. Chest.

[7] Celedón JC (2004). The transforming growth factor-beta1 (TGFB1) gene is associated with chronic obstructive pulmonary disease (COPD). Hum. Mol. Genet.

[8] Chen PY (2016). Fibroblast growth factor (FGF) signaling regulates transforming growth factor beta (TGFβ)-dependent smooth muscle cell phenotype modulation. Sci. Rep..

[9] Lee BJ (2011). Protective effects of basic fibroblast growth factor in the development of emphysema induced by interferon-γ. Exp. Mol. Med..

[10] Bikfalvi A, Klein S, Pintucci G, Rifkin DB (1997). Biological roles of fibroblast growth factor-2. Endocr. Rev..

[11] Nugent MA, Iozzo RV (2000). Fibroblast growth factor-2. Int. J. Biochem. Cell Biol..

[12] Burgess JK (2009). The role of the extracellular matrix and specific growth factors in the regulation of inflammation and remodelling in asthma. Pharmacol. Ther..

[13] Hoshino M, Takahashi M, Aoike N (2001). Expression of vascular endothelial growth factor, basic fibroblast growth factor, and angiogenin immunoreactivity in asthmatic airways and its relationship to angiogenesis. J. Allergy Clin. Immunol..

[14] Redington AE (2001). Basic fibroblast growth factor in asthma: measurement in bronchoalveolar lavage fluid basally and following allergen challenge. J. Allergy Clin. Immunol..

[15] Shute JK (2004). Epithelial expression and release of FGF-2 from heparan sulphate binding sites in bronchial tissue in asthma. Thorax.

[16] Chang SS, Yokomise H, Matsuura N, Gotoh M, Tabata Y (2014). Novel therapeutic approach for pulmonary emphysema using gelatin microspheres releasing basic fibroblast growth factor in a canine model. Surg. Today.

[17] Morino S (2007). Fibroblast growth factor-2 promotes recovery of pulmonary function in a canine models of elastase-induced emphysema. Exp. Lung Res..

[18] Morino S (2005). Fibroblast growth factor-2 induces recovery of pulmonary blood flow in canine emphysema models. Chest.

[19] Kawago M (2014). Intrapleural administration of gelatin-embedded, sustained-release basic fibroblast growth factor for the regeneration of emphysematous lungs in rats. J. Thorac. Cardiovasc. Surg..

[20] Jeon SG (2007). Recombinant basic fibroblast growth factor inhibits the airway hyperresponsiveness, mucus production, and lung inflammation induced by an allergen challenge. J. Allergy Clin. Immunol..

[21] Kim YS (2014). The safety and efficacy of recombinant fibroblast growth factor 2 in human asthmatics: a pilot study. Allergy Asthma Respir. Dis..

[22] Huh JW (2011). Bone marrow cells repair cigarette smoke-induced emphysema in rats. Am. J. Physiol. Lung Cell. Mol. Physiol..

[23] Kim YS (2013). Extracellular vesicles, especially derived from Gram-negative bacteria, in indoor dust induce neutrophilic pulmonary inflammation associated with both Th1 and Th17 cell responses. Clin. Exp. Allergy.

[24] Lazarous DF (1995). Effects of chronic systemic administration of basic fibroblast growth factor on collateral development in the canine heart. Circulation.

[25] Schaper W, De Brabander M, Lewi P (1971). DNA synthesis and mitoses in coronary collateral vessels of the dog. Circ. Res..

[26] Partridge MR (2010). Development and validation of the capacity of daily living during the morning questionnaire and the Global Chest Symptoms Questionnaire in COPD. Eur. Respir. J..

[27] Lommatzsch M (2010). Acute effects of tobacco smoke on human airway dendritic cells in vivo. Eur. Respir. J..

[28] Costabel U, Guzman J (1992). Effect of smoking on bronchoalveolar lavage constituents. Eur. Respir. J..

[29] Kuschner WG, D’Alessandro A, Wong H, Blanc PD (1996). Dose-dependent cigarette smoking-related inflammatory responses in healthy adults. Eur. Respir. J..

[30] Burke WM (1992). Smoking-induced changes in epithelial lining fluid volume, cell density and protein. Eur. Respir. J..

[31] Pardo OE (2002). Fibroblast growth factor-2 induces translational regulation of Bcl-XL and Bcl-2 via a MEK-dependent pathway: correlation with resistance to etoposide-induced apoptosis. J. Biol. Chem..

[32] Pardo OE (2003). Fibroblast growth factor 2-mediated translational control of IAPs blocks mitochondrial release of Smac/DIABLO and apoptosis in small cell lung cancer cells. Mol. Cell Biol..

[33] Aubry MC, Wright JL, Myers JL (2000). The pathology of smoking related lung diseases. Clin. Chest Med..

[34] Sun C (2014). LL-37 secreted by epithelium promotes fibroblast collagen production: a potential mechanism of small airway remodeling in chronic obstructive pulmonary disease. Lab. Invest..

[35] Kim SH (2018). Perceptions of severe asthma and asthma-COPD overlap syndrome among specialists: a questionnaire survey. Allergy Asthma Immunol. Res..

[36] Krimmer DI, Burgess JK, Wooi TK, Black JL, Oliver BG (2012). Matrix proteins from smoke-exposed fibroblasts are pro-proliferative. Am. J. Respir. Cell Mol. Biol..

[37] Hogg JC (2004). The nature of small-airway obstruction in chronic obstructive pulmonary disease. N. Engl. J. Med..

[38] James AL, Wenzel S (2007). Clinical relevance of airway remodelling in airway diseases. Eur. Respir. J..

[39] Turner N, Grose R (2010). Fibroblast growth factor signalling: from development to cancer. Nat. Rev. Cancer.

