# Supplementary material for: The role of FGF-2 in smoke-induced emphysema and the therapeutic potential of recombinant FGF-2 in patients with COPD
Source: Exp Mol Med. 2018 Nov 14;50(11):150. doi: 10.1038/s12276-018-0178-y (PMC6235987; doi:10.1038/s12276-018-0178-y)
Supplement: Supplementary file 1 — Table S1, Table S2, Table S3, Table S4 [file 12276_2018_178_MOESM1_ESM.docx]

**Supplementary Appendix**

**Table S1.** Clinical characteristics of patients for the comparison of plasma FGF-2 concentrations

|  | Subjects | |
| --- | --- | --- |
|  | Normal | COPD |
| Number of subjects | 10 | 10 |
| Age, years | 63 ± 8 | 63 ± 8 |
| Male subjects, % | 100% | 80% |
| Smoking pack-year | 28 ± 7 | 28 ± 7 |
| FEV_1_ % predicted | 95 ± 8 | 33 ± 10 |

FEV_1_ % predicted: forced expiratory volume in 1 s percent (FEV_1_%) of an individual divided by the average FEV_1_% in the population with similar age, sex, and body composition; FGF-2: fibroblast growth factor-2; COPD: chronic obstructive pulmonary disease

**Table S2. Inclusion criteria for the** **clinical trial**

| Out-patient, male or female |
| --- |
| Aged 40–75 years |
| Current or former cigarette smokers with a smoking history ≥ 10 pack-years |
| COPD patients, classified as GOLD II or III in terms of severity, post-bronchodilator FEV1/FVC < 0.7, and FEV1 between 30% and 60% of predicted |
| Patients who use ICS and long-acting bronchodilators (LABAs and/or LAMAs) |

GOLD: Global Initiative for Obstructive Lung Disease; FEV1: forced expiratory volume in 1 s; FVC: forced vital capacity; ICS: inhaled corticosteroid; LABA: long-acting beta 2 agonist; LAMA: long-acting muscarinic antagonist

**Table S3. Exclusion criteria of** **clinical trial**

| Diagnosis of active pulmonary disease, such as pulmonary tuberculosis, bronchiectasis, lung  cancer, respiratory tract infection, or other progressively fatal disease |
| --- |
| Diagnosis of severe cardiovascular disease, such as acute myocardial infarction, arrhythmia,  heart failure, or uncontrolled hypertension |
| Severe neurological disease or severely impaired hepatic or renal function |
| Treatment with systemic corticosteroids or COPD exacerbation within the previous 4 weeks at inclusion |
| Immunocompromised patients |
| Suspected or known hypersensitivity to the study product or any of its excipients |
| Pregnant or lactating women  Poor reliability (e.g., history of alcohol or drug abuse, mental disorder) and poor compliance |
| Patients already enrolled who had received any other investigational drug in the last 3 months prior to study entry |

**Table S4. Adverse events during the clinical trial**

| Adverse event | Control group | FGF-2 group | Severity^a^ |
| --- | --- | --- | --- |
| Back pain | 1 (17%) | 0 (0%) | 1 |
| Cough | 0 (0%) | 2 (33%) | 1 |
| Delirium | 0 (0%) | 1 (17%) | 1 |
| Dyspepsia | 1 (17%) | 1 (17%) | 1 |
| Dyspnea | 1 (17%) | 0 (0%) | 2 |
| Dysuria | 0 (0%) | 1 (17%) | 1 |
| Expectoration discomfort | 0 (0%) | 1 (17%) | 1 |
| General weakness | 0 (0%) | 1 (17%) | 1 |
| Lip discomfort | 0 (0%) | 1 (17%) | 1 |
| Myalgia | 1 (17%) | 0 (0%) | 1 |
| Petechia | 1 (17%) | 0 (0%) | 1 |
| Rhinorrhea | 0 (0%) | 1 (17%) | 1 |
| Sore throat | 0 (0%) | 2 (33%) | 1 |

^a^ 1: mild, 2: moderate, 3: severe, 4: not applicable.

FGF-2: fibroblast growth factor-2.
